# The Efficacy and Cost-Effectiveness of Umeclidinium/Vilanterol versus Tiotropium in Symptomatic Patients with Chronic Obstructive Pulmonary Disease

**DOI:** 10.1155/2022/2878648

**Published:** 2022-08-25

**Authors:** Yinhua Gong, Chen Lin, Yifeng Jin, Rong Chen

**Affiliations:** ^1^Department of Pharmacy, The First Affiliated Hospital of Soochow University, Suzhou, Jiangsu 215006, China; ^2^Suzhou Industrial Park Food and Drug Safety Inspection Team, Suzhou, Jiangsu 215000, China; ^3^Department of General Practice, The First Affiliated Hospital of Soochow University, Suzhou, Jiangsu 215006, China

## Abstract

**Background:**

Both long-acting muscarinic antagonists (LAMAs) and long-acting *β*2-agonists (LABAs) are widely used in the treatment of chronic obstructive pulmonary disease (COPD). A novel LAMA/LABA combination of umeclidinium/vilanterol (UMEC/VI; 62.5 *μ*g/25 *μ*g) is approved for chronic obstructive pulmonary disease (COPD) treatment.

**Objective:**

This study aimed to assess the efficacy and cost-effectiveness of UMEC/VI versus tiotropium (TIO) 18 *μ*g in symptomatic patients with COPD from the perspective of the Chinese National Healthcare System.

**Methods:**

A simple analysis included three studies in the meta-analysis that compared UMEC/VI with TIO. A Markov model was developed to estimate the cost-effectiveness of UMEC/VI compared with TIO treatment in symptomatic patients with COPD. First, utilities, clinical efficacy, and adverse events obtained from the literature were utilized as model inputs. Costs were from Chinese average data, including local data. Costs were expressed in dollars based on 2020 prices. Then, the model outputs including drug costs, other medical costs, and total costs, and quality-adjusted life years (QALYs) were estimated. Costs and outcomes were discounted at a 5% annual rate. Furthermore, incremental cost-effective ratios (ICERs) were analyzed. Finally, the influences of changing parameters on the uncertainty of the results were assessed by means of one-way and probabilistic sensitivity analyses.

**Results:**

This study revealed that UMEC/VI treatment had a higher rate of clinical efficacy in comparison with TIO, and the differences in the rate of adverse events between the two treatments were not significant. The results indicated that UMEC/VI was superior to TIO, which provided an increase in QALYs (0.002) and a total cost savings of $765.67 per patient over 3 years. In the base case, the ICER of UMEC/VI is -$397468.04/QALY compared with TIO, suggesting that UMEC/VI may be considered a dominant option over TIO. According to the Chinese medical system, the probability of UMEC/VI being cost-effective was 61.6% at a willingness-to-pay (WTP) of $31554/QALY. Sensitivity analyses confirmed that the results were robust.

**Conclusion:**

UMEC/VI could be considered a cost-effective treatment compared with TIO in symptomatic COPD patients from the Chinese National Healthcare System perspective. These results may help decision-makers in China when making judgements on which treatments to administer.

## 1. Introduction

Chronic obstructive pulmonary disease (COPD) is a disabling respiratory disease characterized by persistent and progressive airflow limitation. It is a commonly preventable and treatable disease associated with an increased chronic inflammatory response in the lungs to noxious stimuli [1]. It ranks as the fourth leading cause of death worldwide by the World Health Organization (WHO). In China, Chen Wang's Pulmonary Health study [2] indicated that 8.6% of the general Chinese adult population (or 99.9 million Chinese adults) aged 20 years or older in 2015 had spirometry-defined COPD, reaching epidemic proportions. COPD has been a major public health problem and will remain a challenge for clinicians in the 21^st^ century. In Asia, high rates of smoking and air pollution ensure that COPD will continue to pose an ever-increasing public health problem [3] with high healthcare costs. In China, the annual mean health care cost of a COPD patient is approximately $3093.759. Hospitalization and medicine accounted for most of the expenses. COPD is now the third leading cause of death in China, and it is reported that only 2.6% of respondents in China are aware that they have the disease [4].

Inhaled medication is the cornerstone of the pharmacological treatment for patients with asthma and COPD [5]. This drug delivery system has the advantage of delivering the drug directly into the airway and reducing the risk of systemic side effects through high local concentrations [6]. Inhalation of long-acting bronchodilators, which improve lung function and reduce symptoms, is the basis of COPD maintenance treatment. LABA and LAMA can be used alone or in combination. Administration of an LABA + LAMA combination is recommended for patients with severe symptoms or those whose symptoms persist despite treatment. In the USA, the EU, and several other countries, maintenance treatment for COPD consists of LAMA (UMEC) in combination with LABA (VI). Treatment with UMEC/VI, which has a clinically acceptable safety profile, was more effective than treatment with TIO monotherapy or placebo, resulting in improved lung function [7–9]. UMEC/VI inhaled powder aerosol (once daily) has been extensively used in maintenance treatment for COPD since it entered the Chinese market in 2018. A novel fixed-dose combination of LAMA (UMEC) and LABA (VI) (62.5 *μ*g/25 *μ*g) can relieve symptoms in adults with COPD (given once daily) as a maintenance bronchodilator treatment. This study provides a new alternative treatment for symptomatic COPD patients. However, its cost-effectiveness in China remains unknown. Considering the economic burden associated with COPD, drug choice should be based on the expected clinical and economic benefits.

An economic evaluation is considered a valuable tool for drug choice decision-making, especially in a comparative analysis of costs and health outcomes. We should consider the relationship between resource inputs (costs) and intermediate outputs in economic evaluations. Our country has a large population of close to 100 million people with COPD. The national talks are listed in China's medical reform policy. The new variety of UMEC/VI is included in the national talks catalog, which means that more people may use this medicine.

Currently, there is no analysis of the cost-effectiveness of UMEC/VI vs. TIO in China. From the perspective of China's healthcare system, this study aims to evaluate the cost-effectiveness of UMEC/VI vs. TIO for COPD for the first time.

## 2. Methods

A meta-analysis was carried out to estimate the clinical efficacy and safety of UMEC/VI compared with TIO in symptomatic patients with COPD. A cost-effectiveness analysis was performed comparing UMEC/VI with TIO in symptomatic patients with COPD from the perspective of China's healthcare system using data from the literature and clinical studies of UMEC/VI vs. TIO.

### 2.1. Systematic Review

#### 2.1.1. Search Strategy and Study Selection

Searches of Medline, Cochrane Library, ClinicalTrails.gov, China National Knowledge Infrastructure (CNKI) were performed for “Umeclidinium” and “Vilanterol” to collect literature on all randomized clinical trials in humans reported up to 31 January 2020. For each paper selected, a systematic manual search of the bibliographies was carried out. The selected language was limited to English or Chinese.

The criteria for inclusion in the meta-analysis were as follows: (1) study type: prospective cohort studies, randomized controlled trials, and randomized crossover studies; (2) study subjects: patients who were diagnosed with COPD; and (3) interventions:

UMEC/VI (62.5 *μ*g/25 *μ*g) treatment for 24 weeks was compared with TIO (18 *μ*g).

The exclusion criteria in the meta-analysis were inconsistent research topics, drug doses, control drugs, experimental time, and research periods. The process is shown in Supplementary Figure 1.

#### 2.1.2. Data Extraction and Quality Evaluation

Data were independently abstracted from each trial by two researchers; disagreement was resolved by consensus.

The following data were extracted: author and year of publication; the number of clinical trials; study duration; study type; total number of patients; number of intervention groups; number of control groups; average age; sex ratio; GOLD stage classification; current smoker at screening (%); smoking pack-years; trough FEV_1_ on day 169, L; number of patients with on-treatment exacerbation; any on-treatment AEs, *n* (%); on-treatment SAEs; AE reported by 3% of patients in any treatment group, *n* (%).

The quality of the research was assessed according to the Cochrane Collaboration's risk of bias assessment tool. This tool includes several sources of bias, for example, random sequence generation, allocation hiding, blinding of subjects and intervention providers, blinding of result evaluators, incomplete results data, selective results reporting, and other sources of bias.

#### 2.1.3. Data Analysis

The relative risk (RR) and 95% confidence interval (CIs) between interventions were obtained via Review Manager 5.3 software. The random-effects model was used if there was moderate or high heterogeneity between studies (*P* value < 0.1 or *I*^*2*^ value > 50%). There was statistical significance when the *P* value was less than 0.05.

### 2.2. Cost-Effectiveness Model

#### 2.2.1. Model Structure

A Markov model was developed to evaluate the cost-effectiveness of UMEC/VI compared with TIO treatment in symptomatic patients with COPD.

**Figure 1:**
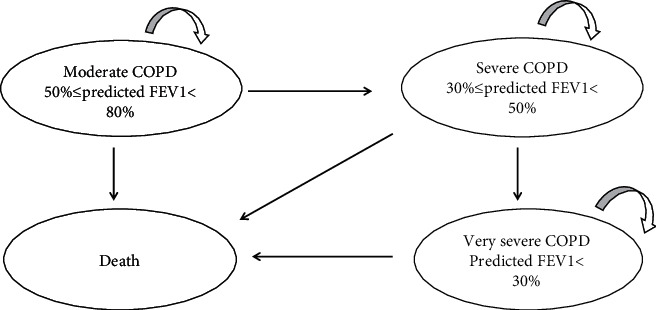
Structure of the decision model used.

According to the three severity levels of COPD defined in the COPD clinical guidelines in 2013, the severity of COPD is classified according to the patient's forced expiratory volume in one second (FEV_1_). There were three disease states (Figure 1): 50% ≤ predicted FEV1 < 80% (moderate COPD), 30% ≤ predicted FEV1 < 50% (severe COPD), predicted FEV1 < 30% (very severe COPD), and death. The cycle length of the Markov model was set to three months [10], as the findings from the NHANES III follow-up study for COPD recommended. The time horizon of the model is three years. After entering the model, patients need to receive maintenance COPD treatment plus routine care. In the model, the patients maintained their current health state of disease severity or moved to the next more serious health state within three months. According to the natural course of the disease, any health condition can lead to death (Figure 1). Cost-effectiveness was then explored through the calculation of incremental cost-effectiveness ratios (ICERs), defined as the difference in mean costs divided by the difference in mean effects. In light of the recommendation by the China Guidelines for Pharmacoeconomic Evaluations 2020 [11], the willingness-to-pay (WTP) for a quality-adjusted life year (QALY) in this study was set to be three times the gross domestic product (GDP) per capita. The average exchange rate of Chinese yuan renminbi for 2020 was 6.899 yuan per US dollar, and the Chinese GDP per capita was $10518 in 2020, according to data from the National Bureau of Statistics(https://www.stats.gov.cn/tjsj/sjjd/202101/t20210119_1812636.html). The WTP threshold was $31554.

#### 2.2.2. Study Perspective

Cost-effective analyses were conducted from the perspective of China's healthcare system, and only direct medical costs were included.

#### 2.2.3. Model Inputs

*(1) Target Population*. Patient characteristics for the base-case analysis were based on three RCTs (NCT01777334, NCT01316913, and NCT01316900) were included in the meta-analysis (Table 1). Eligible patients were administered inhalation therapy of UMEC/VI (62.5 *μ*g/25 *μ*g) for 24 weeks compared with TIO (18 *μ*g) once a day. There was a little difference among the three studies. The method of merging is to merge and calculate according to the proportion of the number of people in each RCT.

*(2) Probabilities*. Each model state comes from the reference, the first cycle uses the initial probability from base to new, and all subsequent cycles use the new probability.

The transitions between the patient's disease severity and health status were based on the method described by Spencer et al., which uses the average patient time in each health status [12].

The transition probabilities of one cycle (3 months) were calculated from the formula: *r* =  − [ln(1 − *P*1)]/*t*1, *P*2 = 1 − exp(−*rt*2); *r* represents the transition rate, and P_1_ and P_2_ represent the transition probabilities for a given cycle length of *t*_1_ and *t*_2_, respectively.

*(3) Efficacy Parameters*. *(Change in Trough FEV*._*1*_) Treatment effects measured by the FEV_1_ for UMEC/VI compared with TIO were obtained from three clinical studies (NCT01777334NCT01316913, and NCT01316900). Trough FEV_1_ at day 169 was the primary endpoint [9]. Meta-analysis was performed after extracting trough FEV_1_ at day 169.

*(4) Exacerbations*. Exacerbations in this model were the events that caused patients to seek healthcare. Patients could experience different levels of exacerbations or no exacerbations. Patients were considered event free if they experienced no exacerbations. Patients who experienced non-severe exacerbations sometimes required a change in treatment, such as antibiotics and/or systemic corticosteroids, and/or contact with doctors. Severe exacerbations required hospitalization. Exacerbation risk was based on patient COPD severity and was obtained from the ECLIPSE study [13]. The number of exacerbations every three months is included in Table 2. Different exacerbation costs were considered in the model.

*(5) Adverse Events (AEs)*. Adverse events were considered in the model. Adverse events occurred in at least 3% of patients and caused significant costs. Adverse events included nasopharyngitis, upper respiratory tract infection, cough, oropharyngeal pain, back pain, and arthralgia, and the corresponding treatment came from expert consultation (Table 3).

*(6) Cost Inputs*. All costs included in the model are in US dollars. The costs included drug costs in China, other medical costs, adverse event costs, and exacerbation costs based on local charges (Table 4). The monthly prescription cost is estimated based on the recommended dose for each treatment. All drug costs are from http://www.yaozh.com, calculated by the median price of the drug in 2020. The corresponding treatment of adverse reactions (usage, dosage, and number of days) is based on expert consultation, and the corresponding treatment cost comes from a tertiary hospital.

*(7) Utility Weights*. The annual utility weight used in the model comes from the literature [12] described by Spencer et al. Estimates of health status by disease stage were generated from the Health Survey for England. This included an assessment of both lung function and health status, measured using the Euro QOL questionnaire (EQ-5D), in 283 patients with COPD.

#### 2.2.4. Cost-Effectiveness of UMEC/VI (62.5 *μ*g/25 *μ*g) Compared with TIO (18 *μ*g)

The disease progression of COPD patients with different therapies was simulated by relying on the model method. The model parameters are shown in Table 2. The cost-effectiveness analysis was used to evaluate the influence of different therapies on patients' long-term quality of life and disease burden. An annual discount rate of 5% was adopted for both effects and costs.

#### 2.2.5. Base-Case Analysis

The cycle length of the Markov model was set to three months [10]. Based on clinical trials and expert opinions, assuming a cycle of 12 weeks (3 months), most patients will change the treatment after three years, so the time horizon of the model is set to three years [15]. The model can also simulate a time span of 1–25 years as needed. Cost and outcomes were discounted at a 5% annual rate in line with Chinese guidelines for economic evaluation [16]. The model estimated costs (total, drug, nondrug, discounted), QALYs gained and incremental cost-effectiveness per QALY gained.

#### 2.2.6. One-Way Sensitivity Analysis

The effect of changing parameters in the model was examined in one-way sensitivity analyses to test the robustness of the model assumptions and specific parameters.

In the one-way sensitivity analysis, the 95% CI of the parameter is preferred as the upper and lower fluctuation range of the parameter [17]. If the 95% CI cannot be obtained, the parameter fluctuation ±20% was used as the fluctuation range.

Data from sources all use 95% CI (such as ADR incidence, utility value), and ±20% (various costs, number of aggravations) are used for sources that cannot be found. The results of the sensitivity analysis for each input were ranked from the most sensitive to the least sensitive and summarized as a tornado diagram, with the 10 most sensitive parameters presented.

#### 2.2.7. Probabilistic Sensitivity Analyses

In the probabilistic sensitivity analysis, the second-order Monte Carlo simulation was used to iterate the model 1,000 times to examine the changes in the results of the base-case analysis when all parameters change in their respective distributions. Based on the results of the second-order Monte Carlo simulation, we constructed cost-effectiveness acceptability curves (CEACs).

## 3. Results

### 3.1. Efficacy and Safety of UMEC/VI (62.5 μg/25 μg) Compared with TIO (18 μg) from Meta-Analysis of Three Clinical Trials

#### 3.1.1. FEV_1_ Results (Efficacy)

Three clinical studies were ultimately included in the meta-analysis after using our search strategy and selection criteria. Trough FEV_1_ at day 169 was the primary endpoint. The selection flow is summarized in Supplementary Figure 1. The basic characteristics of the included RCTs are summarized in Supplementary Table 1. All the included studies were of good methodological quality.

**Figure 2:**
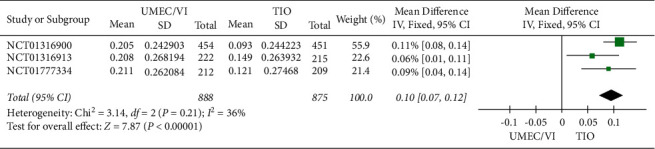
Forest plot of trough FEV_1_ for UMEC/VI vs. TIO using random-effects meta-analysis.

The meta-analysis results (Figure 2) proved that UMEC/VI had higher FEV_1_ results than TIO (RR: 5.96, 95% CI: 2.41–9.51). Based on the results of the meta-analysis, UMEC/VI can significantly increase the FEV_1_ level of COPD patients compared with TIO.

#### 3.1.2. Number of Patients with On-Treatment Exacerbation

**Figure 3:**
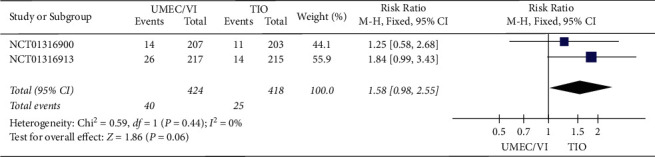
Forest plot of exacerbation rate for UMEC/VI vs. TIO using random-effects meta-analysis.

The analysis (Figure 3) also implied that there was no remarkable difference in the rate of exacerbation between UMEC/VI and TIO treatment (RR: 1.58, 95% CI: 0.98–2.55).

#### 3.1.3. Adverse Events and Severe Adverse Event Results

**Figure 4:**
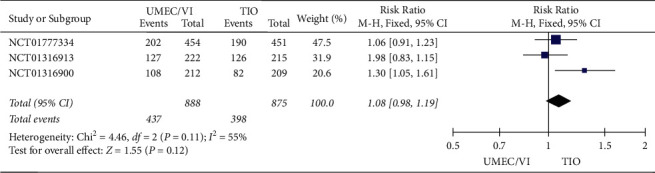
Forest plot of adverse event results for UMEC/VI vs. TIO using random-effects meta-analysis.

*(1) Adverse Event Results*. The analysis (Figure 4) also implied that there was no remarkable difference in the rate of adverse events between UMEC/VI and TIO treatment (RR: 1.08, 95% CI: 0.98–1.19).

No significant difference between UMEC/VI and TIO was observed based on the adverse event results of the meta-analysis.

**Figure 5:**
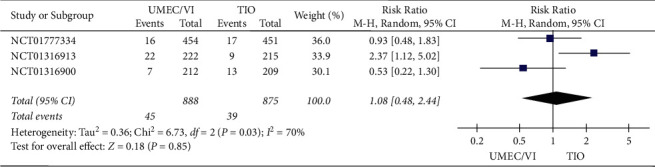
Forest plot of severe adverse event results for UMEC/VI vs. TIO using random-effects meta-analysis.

*(2) Severe Adverse Event Results*. According to the severe adverse event results of the meta-analysis (Figure 5), the difference between UMEC/VI and TIO (RR: 1.08, 95% CI 0.48–2.44) was not statistically significant.

In brief, it could be concluded that UMEC/VI treatment, which did not increase side effects, was more effective than TIO for COPD.

The meta-analysis indicated that not only did UMEC/VI have more significant effects than TIO on improving FEV_1_ (RR: 5.96, 95% CI: 2.41–9.51), but it also did not increase any adverse event results (RR: 1.08, 95% CI: 0.98–1.19) or any severe adverse event results (RR: 1.08, 95% CI 0.48–2.44).

The results of the meta-analysis demonstrated that there was no significant difference between UMEC/VI and TIO in exacerbation rate, incidence of any adverse events or serious adverse events.

### 3.2. Cost-Effectiveness of UMEC/VI vs. TIO

#### 3.2.1. Base-Case Analysis

As shown in Table 5, in comparison with TIO, the use of UMEC/VI to treat COPD was more effective (1.545 QALYs vs. 1.543 QALYs) and cheaper ($5070.82 vs. $5836.49) over a three-year period. The incremental QALY for UMEC/VI compared with TIO was 0.002 QALYs. The incremental cost value for TIO compared with UMEC/VI was 765.67 USD.

In the base case, the ICER of UMEC/VI suggested that UMEC/VI may be considered a superior option to TIO.

#### 3.2.2. One-Way Sensitivity Analysis

The effect of changing parameters was examined in one-way sensitivity analyses to assess the model robustness and the uncertainty of the input parameters. The results of the sensitivity analysis for each input were ranked from the most sensitive to the least sensitive and plotted on a tornado diagram.

**Figure 6:**
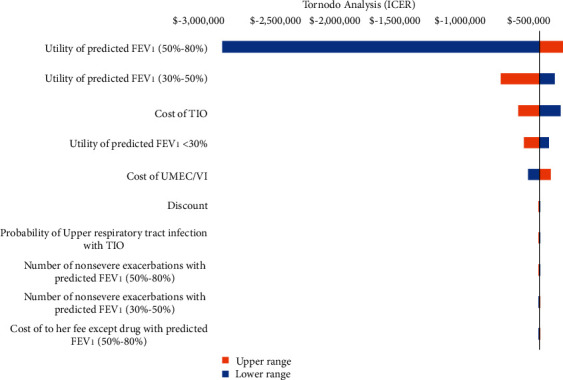
One-way sensitivity analysis. The vertical line in the chart area represents the base ICER value. The width of each bar represents the range of uncertainty associated with each parameter (left of the line: decreased ICER; right of the line: increased ICER).

The utility of predicted FEV_1_ (50%–80%) was identified to have the greatest influence on the results, followed by the utility of predicted FEV_1_ (30%–50%) and the cost of TIO from the results of the one-way sensitivity analysis (Figure 6).

#### 3.2.3. Probabilistic Sensitivity Analysis

Probabilistic sensitivity analyses (second-order Monte Carlo simulation), in which all parameters in the model varied at the same time, were conducted to address the uncertainty in the model input values.

**Figure 7:**
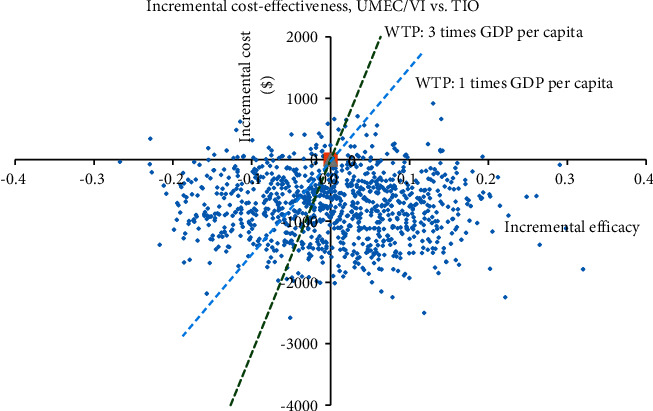
Cost-effectiveness plane for the incremental costs in US dollars compared with the incremental effectiveness in QALYs. Each dot represents a separate run of the model with different input values for each variable randomly selected according to their distribution. According to the Chinese healthcare system, the probability of UMEC/VI being cost-effective was 61.6% at a willingness-to-pay of $31554/QALY.

The ICERs for the 1000 samples in the probabilistic sensitivity analysis (PSA) are shown in the scatter plot (Figure 7).

**Figure 8:**
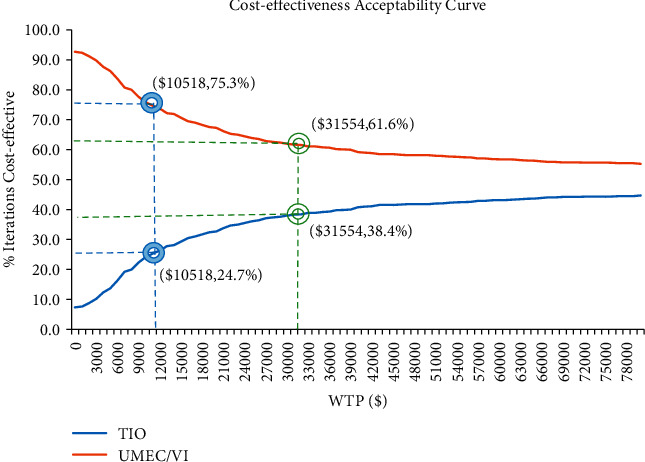
Cost-effectiveness acceptability curve for strategies.

The acceptability curves (Figure 8) further indicated that the cost-effectiveness of UMEC/VI treatment decreased with increasing WTP thresholds, while TIO treatment increased with increasing WTP thresholds.

## 4. Discussion

Not only criteria of efficacy and safety but also cost-effectiveness influences decisions in today's health care environment. Cost-effectiveness analyses can provide information about the economic value of health interventions, often compared to the most commonly used intervention. Cost-effectiveness analyses are tools that can help health managers and decision-makers make informed decisions. The aim of this study was to evaluate the cost-effectiveness of UMEC/VI vs. TIO, the most widely used treatment for COPD. Costs included drug and nondrug expenses, costs related to exacerbation events, and adverse event costs in particular. Including quantity and quality of life and measuring the changes in utility during the patient's life, the QALY is the most acceptable health-related utility measure. As a result of different cumulative numbers of exacerbations over the horizon analyzed, quality of life, percentage of patients with symptoms (dyspnea and cough and sputum), FEV_1_ results and survival, QALY differed between the compared treatments in the model developed.

The analysis originally studied the cost-effectiveness of the combination of two bronchodilators in comparison with monotherapy in China. A similar analysis has been carried out in the UK and Spain. In a UK study, UMEC/VI was considered to be a cost-effective alternative to TIO at a certain price [18]. In a study in Spain, UMEC/VI produced an additional 0.03 QALY and €590 vs. TIO, causing an ICER of €21,475/QALY. According to PSA, the probability of UMEC/VI being cost-effective was 80.3% at a WTP of €30,000/QALY [19]. A cost-effectiveness analysis of indacaterol + glycopyrronium from the perspective of the Swedish NHS supported the efficient use of LAMA + LABA treatment in symptomatic patients [20]. These results are in agreement with previous studies of UMEC/VI and with systematic reviews and meta-analyses of other therapies, which revealed that FEV_1_ benefit is related to improvement in quality of life. The combination of inhaled UMEC with VI has been shown to provide significant improvements in lung function compared with UMEC, VI, or placebo in patients with COPD [21].

Model selection is an important factor in cost-effectiveness analyses, since the model must represent the disease studied. This analysis utilized a published Markov model of disease progression [22]. The model used in COPD is based on pulmonary function for the transition between different health states. Upon entering the model, patients were prescribed maintenance COPD treatment plus usual care. In the model, patients remained in their current disease severity state or moved to the next more severe state each year. Patients could also experience an exacerbation or remain event free. Death could occur from any health state according to the natural progression of the disease. The use of LAMA + LABA combination treatment is recommended in patients with more severe symptoms or those whose symptoms or obvious limitations persist despite receiving monotherapy [23]. The results obtained here support this recommendation, with UMEC/VI being an efficient treatment option compared with TIO monotherapy. In the base-case results, the patients gained more QALYs from UMEC/VI than from TIO.

The cost-effectiveness analysis presented here could be a conservative estimate since some medical costs, such as costs concerning inhaler misuse, were not factored into the model. Poor inhalation techniques can lead to poor disease control, and the costs related to critical errors are considerable [24, 25]. In a recent study, the Ellipta device was compared with other commonly used inhaler devices. Patients who used the Ellipta device had fewer critical errors than those who used the five alternative inhalers [26].

Confirming the results observed within this indirect treatment comparison and comparing the cost-effectiveness of LAMA/LABA vs. LAMA therapies over a longer duration are planned for future studies. Hence, the informed judgements of payers on cost-effectiveness will be needed. Certain assumptions were made in this model about the use of LAMA/LABA combination therapy for a longer duration because data about the timeframes considered in this analysis (3 years, 5 years, 10 years, and lifetime) are not available from current clinical studies. The assumption that there are changes in FEV_1_ once a patient escalates to subsequent triple therapy (LAMA/LABA + ICS) needs to be made due to the lack of data on escalation to triple therapy from LAMA/LABA therapy. It could be a conservative assumption that patients treated with UMEC/VI did not experience a change in FEV_1_ after initiating ICS therapy.

The strength of this study is that it originally studied the cost-effectiveness of the combination of two bronchodilators in comparison with monotherapy in China. In this study, we included the following data: current smoker at screening (%); smoking pack-years; trough FEV_1_ on day 169, L; number of patients with on-treatment exacerbation; any on-treatment AEs, *n* (%); and on-treatment SAEs. Finally, data on baseline SGRQ and 6MWT distances were not available from the sources used for this analysis and had to be estimated within the model. To further facilitate cost-effectiveness evaluations, collecting and including such data in future studies are necessary.

COPD is a chronic progressive condition, and patients with COPD frequently undergo inhalation treatment switches. In the current analysis, we did not consider any inhalation treatment changes. The limitation of these economic evaluations is the quality of the data. In this case, local Chinese data sources were considered. The cost-effectiveness analysis presented here could be a conservative estimate since some medical costs, such as costs concerning inhaler misuse, were not factored in the model. All assumptions were validated by the researchers, and a sensitivity analysis was performed to evaluate uncertainty. Although Chinese costs were used in these sensitivity analyses, we nevertheless provide the most applicable platform by which to assess the cost-effectiveness of UMEC/VI vs. TIO from the perspective of other nations using nationally derived cost inputs.

## 5. Conclusion

The results of this cost-effectiveness analysis show that treatment with UMEC/VI in symptomatic COPD patients is a cost-effective option compared with TIO from the perspective of the Chinese NHS, as the ICER was below the threshold commonly accepted in China to consider interventions as efficient.

## Figures and Tables

**Table 1:** Baseline characteristics of the population included in the analysis.

Characteristic		UMEC/VI (*N* = 888)	TIO (*N* = 875)
Age (years), mean (SD)		62.94 (8.62)	63.29 (8.73)
Male (%)		67.34	68.11
Current smoker at screening, *n* (%)		51.8	50.74
Smoking pack-years, mean (SD)		45.19 (25.68)	46.16 (27.02)
GOLD stage	II, *n* (%)	395 (44.48)	389 (44.46)
	III, *n* (%)	375 (42.23)	376 (42.97)
	IV, *n* (%)	111 (12.50)	106 (12.11)

**Table 2:** Model parameters.

Input parameter	Value	Distribution	Distribution parameter	
			*α*	*β*
Outcome probabilities				
P (A ⟶ A)-UMEC/VI	0.952	Dirichlet	1234	62
P (A ⟶ B)-UMEC/VI	0.000	Dirichlet	0	1296 [15]
P (A ⟶ C)-UMEC/VI	0.000	Dirichlet	0	1296
P (A ⟶ death)-UMEC/VI	0.048	Dirichlet	62	1234
P (B ⟶ A)-UMEC/VI	0.030	Dirichlet	39	1257
P (B ⟶ B)-UMEC/VI	0.922	Dirichlet	1195	101
P (B ⟶ C)-UMEC/VI	0.000	Dirichlet	0	1296
P (B ⟶ death)-UMEC/VI	0.048	Dirichlet	62	1234
P (C ⟶ A)-UMEC/VI	0.000	Dirichlet	0	1296
P (C ⟶ B)-UMEC/VI	0.030	Dirichlet	39	1257
P (C ⟶ C)-UMEC/VI	0.944	Dirichlet	1223	73
P (C ⟶ death)-UMEC/VI	0.026	Dirichlet	34	1262
P (A ⟶ A)-new-UMEC/VI	0.952	Dirichlet	1234	62
P (A ⟶ B)-new-UMEC/VI	0.000	Dirichlet	0	1296
P (A ⟶ C)-new-UMEC/VI	0.000	Dirichlet	0	1296
P (A ⟶ death)-new-UMEC/VI	0.048	Dirichlet	62	1234
P (B ⟶ A)-new-UMEC/VI	0.043	Dirichlet	56	1240
P (B ⟶ B)-new-UMEC/VI	0.909	Dirichlet	1178	118
P (B ⟶ C)-new-UMEC/VI	0.000	Dirichlet	0	1296
P (B ⟶ death)-new-UMEC/VI	0.048	Dirichlet	62	1234
P (C ⟶ A)-new-UMEC/VI	0.000	Dirichlet	0	1296
P (C ⟶ B)-new-UMEC/VI	0.026	Dirichlet	34	1262
P (C ⟶ C)-new-UMEC/VI	0.948	Dirichlet	1228	68
P (C ⟶ death)-new-UMEC/VI	0.026	Dirichlet	34	1262
P (A ⟶ A)-TIO	0.952	Dirichlet	832	42
P (A ⟶ B)-TIO	0.000	Dirichlet	0	874 [15]
P (A ⟶ C)-TIO	0.000	Dirichlet	0	874
P (A ⟶ death)-TIO	0.048	Dirichlet	42	832
P (B ⟶ A)-TIO	0.041	Dirichlet	36	838
P (B ⟶ B)-TIO	0.911	Dirichlet	796	78
P (B ⟶ C)-TIO	0.000	Dirichlet	0	874
P (B ⟶ death)-TIO	0.048	Dirichlet	42	832
P (C ⟶ A)-TIO	0.000	Dirichlet	0	874
P (C ⟶ B)-TIO	0.038	Dirichlet	33	841
P (C ⟶ C)-TIO	0.936	Dirichlet	818	56
P (C ⟶ death)-TIO	0.026	Dirichlet	23	851
P (A ⟶ A)-new-TIO	0.952	Dirichlet	832	42
P (A ⟶ B)-new-TIO	0.000	Dirichlet	0	874
P (A ⟶ C)-new-TIO	0.000	Dirichlet	0	874
P (A ⟶ death)-new-TIO	0.048	Dirichlet	42	832
P (B ⟶ A)-new-TIO	0.043	Dirichlet	38	836
P (B ⟶ B)-new-TIO	0.909	Dirichlet	794	80
P (B ⟶ C)-new-TIO	0.000	Dirichlet	0	874
P (B ⟶ death)-new-TIO	0.048	Dirichlet	42	832
P (C ⟶ A)-new-TIO	0.000	Dirichlet	0	874
P (C ⟶ B)-new-TIO	0.026	Dirichlet	23	851
P (C ⟶ C)-new-TIO	0.948	Dirichlet	829	45
P (C ⟶ death)-new-TIO	0.026	Dirichlet	23	851

ADR				
P back pain-UMEC/VI	1.54%	*γ*	21.6629	0.0007
P cough-UMEC/VI	1.43%	*γ*	142.2286	0.0001
P headache-UMEC/VI	4.70%	*γ*	1803.471	0.0000
P nasopharyngitis-UMEC/VI	3.63%	*γ*	50.4199	0.0007
P upper respiratory tract infection-UMEC/VI	1.65%	*γ*	123.5419	0.0001
P back pain-TIO	1.61%	*γ*	16.8545	0.0010
P cough-TIO	1.50%	*γ*	183.1319	0.0001
P headache-TIO	3.20%	*γ*	77.0869	0.0004
P nasopharyngitis-TIO	3.67%	*γ*	499.4234	0.0001
P upper respiratory tract infection-TIO	2.64%	*γ*	44.4837	0.0006

Cost				
Drug cost				
C UMEC/VI	$95.223	Uniform	76.1783	114.2675
C TIO	$180.528	Uniform	144.4223	216.6334

Other medical costs				
C predicted FEV_1_ (50%–80%)	$143.370	Uniform	114.6958	172.0437
C predicted FEV_1_ (30%–50%)	$204.289	Uniform	163.4312	245.1468
C predicted FEV_1_ (<30%)	$319.454	Uniform	255.5629	383.3443

Adverse event cost				
C back pain	$3.189	Uniform	2.5509	3.8263
C cough	$32.196	Uniform	25.7569	38.6353
C headache	$3.189	Uniform	2.5509	3.8263
C nasopharyngitis	$3.189	Uniform	2.5509	3.8263
C oropharyngeal pain	$15.972	Uniform	12.7776	19.1663
C upper respiratory tract infection	$39.214	Uniform	31.3711	47.0566

Exacerbation cost				
C severe exacerbation	$2,129.690	Uniform	1703.7522	2555.6283
C nonsevere exacerbation	$44.824	Uniform	35.8594	53.7891

Utility				
U predicted FEV1 (<30%)	0.670	*β*	58.5848	28.8552
U predicted FEV_1_ (30%–50%)	0.720	*β*	160.5600	62.4400
U predicted FEV_1_ (50%–80%)	0.810	*β*	310.8375	72.9125
Discount (quarterly)	0.01			

Exacerbations				
Number of severe exacerbations with predicted FEV_1_ (50%–80%)	0.028 (13)	Uniform	0.0220	0.0330
Number of severe exacerbations with predicted FEV_1_ (30%–50%)	0.063	Uniform	0.0500	0.0750
Number of severe exacerbations with predicted FEV_1_ (<30%)	0.135	Uniform	0.1080	0.1620
Number of nonsevere exacerbations with predicted FEV_1_ (50%–80%)	0.185	Uniform	0.1480	0.2220
Number of nonsevere exacerbations with predicted FEV_1_ (30%–50%)	0.273	Uniform	0.2180	0.3270
Number of nonsevere exacerbations with predicted FEV_1_ (<30%)	0.365	Uniform	0.2920	0.4380

In the calculation, the quarterly discount was 1%. The number of different severe exacerbations (13) was the average number of seizures in a patient over a three-month period. P: probability, C: cost, U: utility. Different status: predicted FEV1 (<30%): A, predicted FEV1 (30%–50%): B, predicted FEV1 (50%–80%): C.

**Table 3:** Adverse event rates of UMEC/VI and TIO.

	UMEC/VI	TIO	Source/assumption
*N*	888	875	NCT01777334, NCT01316913, NCT01316900
Back pain	3.43%	3.30%	
Cough	2.81%	2.84%	
Headache	9.23%	6.05%	
Nasopharyngitis	7.46%	7.40%	
Upper respiratory tract infection	3.24%	5.17%	

**Table 4:** Treatment-related costs of chronic obstructive pulmonary disease.

Input parameter	Value	Source/assumption
Drug costs	Quarterly prescription costs (calculated based on the winning bid price in 2020)	
UMEC/VI	$95.22	http://www.yaozh.com
TIO	$180.53	http://www.yaozh.com

Other medical costs	Quarterly costs	
Moderate COPD	$143.37	Reference [14]
Severe COPD	$204.29	
Very severe COPD	$319.45	Physician visit: local charge

Adverse events (AEs)	Costs a (per reported AE)	Physician visit and local processing charge
Back pain	$3.19	Physician visit: local charge
Cough	$32.2	Physician visit: local charge
Headache	$3.19	Physician visit and local processing charge
Nasopharyngitis	$3.19	
Upper respiratory tract infection	$39.21	

Exacerbation cost		
Severe exacerbation	$2,129.690	
Nonsevere exacerbation	$44.824	

**Table 5:** Costs and effectiveness of UMEC/VI versus TIO in the base case analysis over a three-year period.

Strategy	Cost (US$)	Effectiveness (QALYs)	Increase cost (US$)	Increase QALY (QALYs)	ICER (US$/QALYs)
TIO	5836.49	1.543	—	—	—
UMEC/VI	5070.82	1.545	−765.67	0.002	Dominant

## Data Availability

The data used to support the findings of this study are included within the article.

## Abbreviations

COPD: Chronic obstructive pulmonary disease
UMEC/VI: Umeclidinium bromide/vilanterol
TIO: Tiotropium
CI: Confidence interval
QALYs: Quality-adjusted life years
AE: Adverse events
SAE: Severe adverse events
RR: Relative risk
GDP: Gross domestic product
EQ-5D: EuroQol-5 dimension questionnaire
FEV_1_: Forced expiratory volume in 1 s
GOLD: Global initiative for chronic obstructive lung disease
ICS: Inhaled corticosteroid
LABA: Long-acting *β*2-agonist
LAMA: Long-acting muscarinic antagonist
ECLIPSE: Evaluation of COPD longitudinally to identify predictive surrogate endpoints
CEACs: Constructed cost-effectiveness acceptability curves
PSA: Probabilistic sensitivity analyses
WTP: Willingness-to-pay
ICER: Incremental cost-effectiveness ratio
6MWT: 6-min walk test
mMRC: Modified medical research council
SGRQ: St. George's Respiratory Questionnaire for COPD patients
NHS: National Health Service.

## Supplementary Materials

Supplementary Materials Searches of Medline, Cochrane Library, ClinicalTrails.gov, and China National Knowledge Infrastructure (CNKI) were performed for “Umeclidinium” and “Vilanterol” to collect literature on all randomized clinical trials in humans reported up to 31 January 2020. The process is shown in Supplementary Figure 1. The selection flow is summarized in Supplementary Figure 1. The basic characteristics of the included RCTs are summarized in Supplementary Table 1.
