# Supplementary material for: The Efficacy and Cost-Effectiveness of Umeclidinium/Vilanterol versus Tiotropium in Symptomatic Patients with Chronic Obstructive Pulmonary Disease
Source: Can Respir J. 2022 Aug 25;2022:2878648. doi: 10.1155/2022/2878648 (PMC9436597; doi:10.1155/2022/2878648)
Supplement: Supplementary Materials — Searches of Medline, Cochrane Library, ClinicalTrails.gov, and China National Knowledge Infrastructure (CNKI) were performed for “Umeclidinium” and “Vilanterol” to collect literature on all randomized clinical trials in humans reported up to 31 January 2020. The process is shown in Supplementary Figure 1. The selection flow is summarized in Supplementary Figure 1. The basic characteristics of the included RCTs are summarized in Supplementary Table 1. [file 2878648.f1.zip › Supplementary Table1.pdf]

Table 1 Basic characteristics of the included RCTs

Year	2014	2017/2014	2017/2014
Author	Maleki-Yazdi, MR	Maleki-Yazdi MR /Decramer M	Maleki-Yazdi MR /Decramer M
Clinical trial number	NCT01777334	NCT01316913	NCT01316900
Study time	24-week	24-week	24-week
Total patients	789/905	341/437	358/421
Intervention group	UMEC/VI 62.5/25 μg	UMEC/VI 62.5/25 μg	UMEC/VI 62.5/25 μg
Number in intervention group	401/454	165/222	181/212
Control group	TIO	TIO	TIO
Number in control group	388/451	176/215	177/209
Average age (mean± SD)	61.9 (8.41)<br>62.7 (8.50)	65.0(8.62)<br>65.2(8.30)	63.0 (8.7)<br>62.6 (9.4)
Gender (male)n	310<br>303	140<br>153	148<br>140
GOLD Stage			
1	/	/	/
2	185 (41%)<br>190 (42%)	106 (49%)<br>103 (48%)	104 (49%)<br>96 (46%)
3	207 (46%)<br>206 (46%)	83 (38%)<br>83 (39%)	85 (40%)<br>87 (42%)
4	62 (14%)<br>55 (12%)	27 (12%)<br>28 (13%)	22 (10%)<br>23 (11%)
Current smoker at screening n (%)	270 (59%)<br>243 (54%)	92 (42%)<br>102 (47%)	98 (46%)<br>99 (48%)
Smoking pack-years Mean (SD)	44.1 (24.44)<br>44.4 (25.03)	47.8 (26.1)<br>54.0 (31.6)	44.8 (27.7)<br>41.9 (24.4)
Patients with at least one COPD exacerbation in the previous year			
Required oral or systemic corticosteroids, antibiotics, or both (without hospital admission)	/<br>/	60 (28%)<br>66 (31%)	64 (30%)<br>70 (34%)
Required hospital admission	/<br>/	9 (4%)<br>14 (7%)	31 (15%)<br>39 (19%)
