# Supplementary figures and images for: The Efficacy and Cost-Effectiveness of Umeclidinium/Vilanterol versus Tiotropium in Symptomatic Patients with Chronic Obstructive Pulmonary Disease
Source: Can Respir J. 2022 Aug 25;2022:2878648. doi: 10.1155/2022/2878648 (PMC9436597; doi:10.1155/2022/2878648)

Fig 1. The trial selection flow

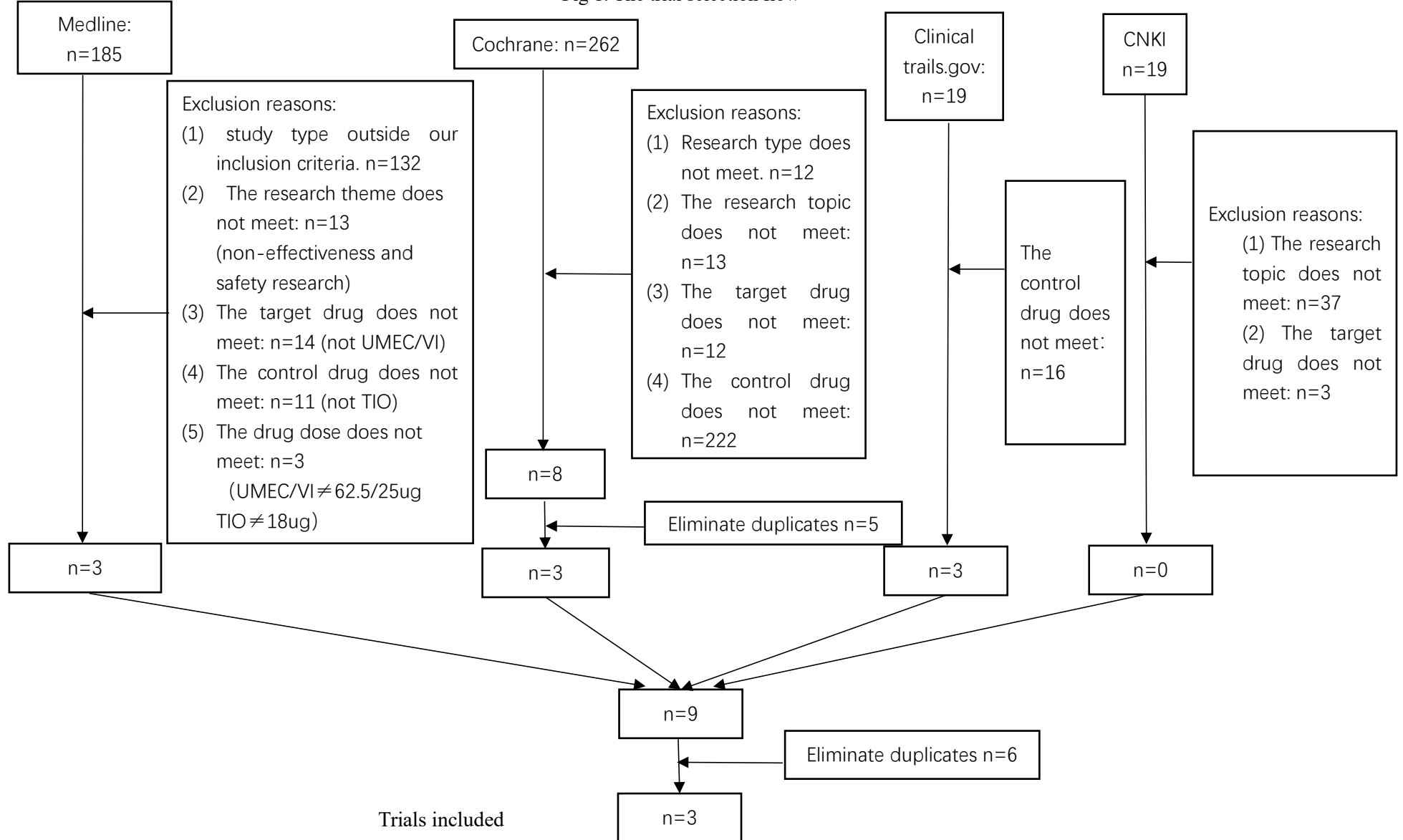

Supplement: Supplementary Materials — Searches of Medline, Cochrane Library, ClinicalTrails.gov, and China National Knowledge Infrastructure (CNKI) were performed for “Umeclidinium” and “Vilanterol” to collect literature on all randomized clinical trials in humans reported up to 31 January 2020. The process is shown in Supplementary Figure 1. The selection flow is summarized in Supplementary Figure 1. The basic characteristics of the included RCTs are summarized in Supplementary Table 1. [file 2878648.f1.zip › Supplementary Figure 1 (1).pdf]
